# Mycotoxin binder for immune and intestinal histopathology ameliorations against Newcastle disease in vaccinated broilers

**DOI:** 10.12688/f1000research.158103.2

**Published:** 2025-01-27

**Authors:** Erma Safitri, Hery Purnobasuki, Tita Damayanti Lestari, Suzanita Utama, Merisa Wahyu Erdhina, Maulida Ilma Sadida, Eka Pramyrtha Hestianah, Mohammad Anam Al Arif, Chairul Anwar Nidom, Sri Mulyati, Jola Rahmahani, Martia Rani Tacharina, Sri Hidanah, Siti Darodjah Rasad, Goo Jang, Mitsuhiro Takagi, Muhammad Thohawi Elziyad Purnama

**Affiliations:** 1 Department of Veterinary Science, Division of Veterinary Reproduction, Faculty of Veterinary Medicine, Universitas Airlangga, Surabaya, East Java, 60115, Indonesia; 2 Department of Biology, Faculty of Science and Technology, Universitas Airlangga, Surabaya, East Java, 60115, Indonesia; 3 Undergraduate Student, Faculty of Veterinary Medicine, Universitas Airlangga, Surabaya, East Java, 60115, Indonesia; 4 Department of Veterinary Science, Division of Veterinary Anatomy, Faculty of Veterinary Medicine, Universitas Airlangga, Surabaya, East Java, 60115, Indonesia; 5 Department of Veterinary Science, Division of Animal Husbandry, Faculty of Veterinary Medicine, Universitas Airlangga, Surabaya, East Java, 60115, Indonesia; 6 Department of Veterinary Science, Division of Basic Veterinary Medicine, Faculty of Veterinary Medicine, Universitas Airlangga, Surabaya, East Java, 60115, Indonesia; 7 Department of Veterinary Science, Division of Veterinary Microbiology, Faculty of Veterinary Medicine, Universitas Airlangga, Surabaya, East Java, 60115, Indonesia; 8 Department of Animal Production, Faculty of Animal Science, Universitas Padjadjaran, Sumedang, West Java, 45363, Indonesia; 9 Department of Theriogenology, College of Veterinary Medicine, Seoul National University, Gwanak-gu, Seoul, 151-742, South Korea; 10 Laboratory of Theriogenology, Joint Faculty of Veterinary Medicine, Yamaguchi University, Yamaguchi, Yamaguchi Prefecture, 753-8515, Japan; 11 Department of Biology, Graduate School of Natural and Applied Sciences, Eskisehir Osmangazi Universitesi, Eskişehir, Eskişehir Province, 26040, Turkey

**Keywords:** aflatoxin, ochratoxin, antibody titer, broiler, toxin binders, food production

## Abstract

**Background:**

In broiler farming, vaccination against Newcastle disease (ND) is essential. Nonetheless, during the post-vaccination phase, production may be negatively impacted by mycotoxin contamination in feed. This study aimed to evaluate the effect of mycotoxin binders on immune and intestinal histopathology ameliorations against newcastle disease in vaccinated broilers with aflatoxin B1 (AFB1) and ochratoxin A (OTA) toxication.

**Methods:**

A total of 20 broilers were randomly assigned into 4 groups with 5 replications i.e. (C-) broiler groups with basal feed, (C+) broiler groups with AFB1 and OTA feed contamination, (T1) and (T2) broiler groups with exposed AFB1, OTA, and toxin binders as feed additives with dose 1.1 g/kg and 1.6 g/kg feed, respectively. ND vaccination was carried out on day 7 and 21. Antibody titers were evaluated from serum samples of broiler on days 14, 28, and 35 for further hemagglutination inhibition (HI) test. Histopathology of the cecum and colon organs was evaluated using HE staining on day 36. HI test and histological scoring were analyzed using the One-Way ANOVA, followed by Duncan’s test with a p < 0.05 in SPSS v.26 software (IBM Corp., Armonk, NY).

**Results:**

As a result, histopathological improvement of the cecum and colon was reported based on mucosal rupture, hemorrhage and necrosis on day 35. An increase in the mean antibody titer compared to days 14 and 28 was observed on day 35, with significant changes observed in serum samples based on the C+ group, which was significantly different from the C- and T2 groups (p < 0.05).

**Conclusions:**

This study revealed that a 1.6 g/kg toxin binder dose in feed can increase antibody titer and histopathology of cecum and colon in broiler chickens after ND vaccination fed with mycotoxin-contaminated feed.

## Introduction

The presence of immunosuppressant substances, such as mycotoxins including aflatoxins B1 (AFB1) and ochratoxins A (OTA), ^1^ and in tandem are the mycotoxin combination that has been shown to have high toxicity. ^2^ Which can decrease the body’s immune response to vaccination, may be the reason for antibody titer findings that indicate no protection against vaccination. ^3^ Immunosuppressive disorders result from T cell proliferation failure carried on by immunotoxic processes, which prevents the body from responding to the incoming vaccination. ^4^^,^^5^ In the short term, OTA and AFB1 interacting to contaminate chicken feed will provide a synergistic negative effect. ^6^ The digestive system has the highest risk of exposure since animal feed is the first point in the food chain where mix mycotoxin can enter directly through plant goods like grains. ^7^ The process of digesting and absorbing dietary fibre in chickens is significantly aided by the large intestine. ^8^^,^^9^

Ochratoxin A profoundly alters the microbiota’s composition and metabolism in the large intestine, which has an impact on the process of fermentative digestion that occurs there. ^10^ Aflatoxin B1 plays a significant role in the production of reactive oxygen species (ROS), which damage DNA and cause lesions in the mitochondria. ^11^^,^^12^ Damage to the membranes and nuclear structures of cells can result in necrosis or death of the cells. ^13^ Mycotoxicosis is a poisoning that happens when an animal eats feed that is too toxic for their body to tolerate. When mix mycotoxin is exposed to the large intestine, it first disrupts the synthesis and breakdown of proteins, which alters the integrity of the intestine and damages the intestinal mucosa. Mix mycotoxin exposure will eventually cause oxidative damage. Large volumes of reactive oxygen species (ROS) are released that can injuring tissue and inhibit tissue repair, ultimately leading to cell necrosis. ^14^ In the gastrointestinal system, mycotoxin binders generally act as adsorbents. One form of adsorbent that has been extensively researched in vivo and in vitro that is effective at removing mycotoxins from the body is bentonite ( *Dioctahedral montmorillonite*). Because the fluid in the gastrointestinal system has a negative charge on its surface, bentonite will be distributed there and utilise cation exchange processes to absorb different biomolecules, including aflatoxin. ^15^ Mycotoxin binder is a big molecular molecule that resembles a chemical sponge and is used to bind mixtures of mycotoxin in an animal’s digestive tract before it is expelled through its faeces. Mycotoxin binders have the ability to reduce the amount of mix mycotoxin in feed, which in turn lessens the amount of mix mycotoxin that penetrates the bloodstream and other bodily organs and causes mycotoxicosis. ^16^ Mycotoxin binders in this research, also known as biotransforming agents, with different function in that they alter the chemical makeup of mycotoxins to be non-toxic molecules. This biotransforming agent was classified as yeast ( *Trichosporon mycotoxinivorans*). As a biotransforming agent, the aforementioned yeast can hydrolise amide bonds in the chemical structure of OTA to break it down into new chemical and non-toxic molecules. ^17^ Vaccination against Newcastle disease (ND) in broiler farming is essential. Nonetheless, during the post-vaccination phase, production may be negatively impacted by mycotoxin contamination in feed.

This study aimed to evaluate the effect of mycotoxin binders on immune and intestinal histopathology ameliorations against Newcastle disease in vaccinated broilers with aflatoxin B1 (AFB1) and ochratoxin A (OTA) toxication.

## Methods

### Ethics statement

This study was approved by the Ethical Committee of Animal Care and Use, Faculty of Veterinary Medicine, Universitas Airlangga (No. 1.KEH.033.02.2023) on February 10, 2023. This study was also reported according to the Animal Research: Reporting of in vivo Experiments (ARRIVE) guidelines 2.0: author checklist. ^40^ All efforts were made to ameliorate any suffering of animals.

### Study period and location

All experiments in this study were conducted from February 27 to June 10, 2023 in an animal laboratory. Antibody titer evaluation was conducted in the microbiology laboratory, while histopathology evaluation was conducted in the pathology laboratory, Faculty of Veterinary Medicine, Universitas Airlangga.

### Experimental animals

The five steps of preparation before acclimatization of day-old chicks (DOC) are as follows: 1. Clean and remove all physical materials from a chicken cage; 2. Wash and clean all equipment and the cage with soap and water, using scraping; 3. Spray liquid disinfectant on all equipment and the cage and close the cage for a week; 4. Spray solid disinfectant (lime) and mix it with water in the cage and close the cage for a week; 5. Fumigate with permanganate and formalin, close the cage, as this can irritate; and finally, wait a week before DOC arrives.

A total of 20 DOC broiler chickens Cobb strain were reared for 35 days as final stock following broiler rearing standards. Rearing standards for broiler experiments include: basic ventilation system (open house), rodent and flyproof, cleaning and disinfection protocol (five steps before DOC arrived), ambient temperature (18 to 24°C), relative humidity (50 to 70%), manual feeding system, watering system (manual, ad libitum, and groundwater), lighting program (light color at 20 lux intensity for the first 7 days and 17-20 hours duration), and lighting program. During rearing, the chickens were fed with a standard formulation for starter-stage broilers with the brand Hipprovite CP511 (Cat No. CP511-511, PT. Charoen Pokphand, Indonesia) and provided drinking water ad libitum. They were assigned into 4 treatment groups and 5 replications, i.e. (C-) was fed basal feed, (C+) was fed with AFB1 and OTA contamination, (T1) and (T2) were fed with AFB1 and OTA contamination and toxin binders (Mycofix ^®^, Cat No. 20026289-FTS, PT. Biomin Indonesia) as feed additives with doses 1.1 g/kg and 1.6 g/kg feed, respectively.

ND vaccination was performed on seven-day-old broilers using La Sota inactivated ND vaccine (Medivac ^®^, Cat No. 3526004001, PT. Medion Farma, Indonesia) via eye drops at a dose of 1 drop/head. Then 21-day-old broilers were booster vaccinated using the inactive ND Medivac ^®^ vaccine at a dose of 0.5 mL/head intramuscularly. The antibody titers were evaluated from serum samples on days 14, 28, and 35 for the hemagglutination inhibition (HI) test. The cecum and colon organs were collected on day 36 to evaluate histopathology using hematoxylin-eosin (HE) staining.

### Hemagglutination (HA) and hemagglutination inhibition (HI) tests

Erythrocytes suspension was collected from ND virus antibody-negative donor chickens. Blood was drawn from the brachial vein and collected in tubes containing EDTA solution (BD Vacutainer ^®^ EDTA Tubes, Cat No. 366643, USA). The blood was washed thrice using PBS by centrifuging at 1.500 *g* for 10 minutes. The buffy coat and plasma fluid were discarded at each washing. The erythrocyte residue was then diluted with PBS to a concentration of 0.5%.

A total of 1 mL blood samples without anti-coagulant were collected through the brachial vein in 20 broiler chickens aged 14, 28, and 35 days. Blood was then allowed until collected serum and transferred into an Ependorf tube. The HA test was performed to determine the antigen titer that will be used for the HI test. This test begins with filling the microplate wells (A1–A12) with 25 μl of PBS. Furthermore, 25 μl of antigen was inserted into the A1 and A12 wells (antigen control). The antigen and PBS in the A1 well were then homogenized with the suction-blow technique using a 25 μl micropipette, then 25 μl of liquid was transferred from one well to the next well, and so on until it reached the A11 well. The remaining 25 μl in the micropipette was discarded. Furthermore, all A1–A12 wells were filled with 50 μl of 0.5% chicken erythrocyte suspension. The microplate was then shaken and incubated at room temperature for 30 minutes, and the titer was ready to evaluate.

The microplate was filled with 25 μl of PBS solution into number 1–5 wells in rows A and B (duplicate titration) using a micropipette. Then, the first well in rows A and B was filled with 25 μl of ND antigen. Antigen and PBS in 1 well were then homogenized by the suction-blowing technique using a 25 μl micropipette, then 25 μl of liquid was transferred from one well to the next well, and so on until reaching the 4 wells. Furthermore, all wells were filled with 50 μl of 0.5% chicken erythrocyte suspension. The microplate was then shaken and incubated at room temperature for 30 minutes, and the titer was ready to interpret.

The first until the twelfth microplate wells were filled with 25 μl of PBS using a micropipette. Then, the first and twelfth wells are filled with 25 μl of serum to be checked for titer. Serum and PBS were mixed in the first well by the suction-blowing technique using a micropipette, then 25 μl of liquid was transferred from the first well to the next well, and so on until the tenth well. Next, wells 1–10 were filled with 25 μl of ND antigen. The microplate was then shaken and incubated for 30 minutes at room temperature. Next, all wells were filled with 50 μl of 0.5% chicken erythrocyte suspension and incubated for 30 minutes for further evaluation.

### Euthanasia in animals

On the 36th day, the cervical dislocation procedure was used to perform euthanasia. ^18^ All efforts were made to ameliorate any suffering of animals. In the event that the study’s chickens displayed signs of illness prior to the termination time, they received vitamin supplements to enhance their health and were treated in accordance with the disease’s symptoms. Nevertheless, the deceased chicken was taken out and insinerated if it died before the termination time.

The procedure of manual cervical dislocation (CD) involves twisting and stretching the neck with the hands. After being picked up, the chickens were fed to help them relax. As we gradually repositioned our hold, we waited for the chickens to calm down. The non-dominant hand was used to grasp the chicken legs. We slowly turned the chicken so that its feet were pointing up at our chest. To keep them motionless, the chicken feet were securely held close to the base of their rear end. Grasping the upper part of the chicken’s neck, where the soft, spongy vertebrae meet the brain stem, fingers were wrapped around them. Gently, the thumb and index finger were encircling this section of the chicken’s neck. After that, the neck of the fowl was angled 90 degrees downward. The chicken’s body was gradually moved to position its head away from the dominant side. Their feet were securely grasped while the head and neck of the chicken were arranged in an L configuration, the beak pointing downward. The twisting motion dislocates the vertebral column from the skull and ruptures or stretches the blood vessels, while the stretching action tears the neck muscles, ligaments, connective tissues, and blood vessels. The cessation of the pupillary light reflex and nictitating membrane reflex were death indicators in broiler chickens. ^18, 19^ The process of incineration involves burning carcasses or residues at temperatures as high as 850 °C to create an inorganic ash. All infectious agents are anticipated to be destroyed by the treatment. Ash usually makes about 1–5% of the initial volume of the carcass, though this will vary depending on the type of incinerator, the method, the fuel, and the species of animal. ^20^

### Histopathological evaluation

Sample collection was performed through abdominal dissection after euthanization. After the cecum and colon from 20 broilers were collected, the organs were fixed in pot containers using a 10% formalin solution to preserve histopathological preparations using hematoxylin-eosin (HE) staining. The histopathological evaluation was performed using a microscope (Nikon Eclipse E200, Japan) in five fields of view to investigate rupture, hemorrhage, and necrosis. The scoring method used the following criteria, (0) no change, (1) <25% pathological findings, (2) 26–50% pathological findings, (3) 51–75% pathological findings, and (4) >76% pathological findings. ^21^

### Statistical analysis

All data were tabulated and then tested for normality using the Shapiro Wilk test. Normally distributed data were followed by One-Way analysis of variance (ANOVA) and Duncan’s multiple comparison post hoc test. All analyses were evaluated using SPSS v.26 for Windows (IBM Corp., Armonk, NY) with decision considerations using a 95% confidence level.

## Results

### Hemagglutination inhibition (HI) test

Results in this research have increased in the mean antibody titer observed on day 35 compared to days 14 and 28, with significant changes observed in serum samples based on the C+ group, which was significantly different from the C- and T2 groups (p < 0.05).

**Figure 1.**
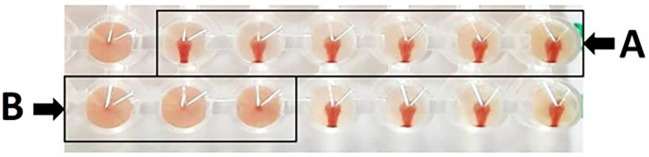
Hemagglutination inhibition test on microplate, with (A) positive HI test, and (B) negative HI test.

Based on hemagglutination inhibition (HI) test, as a result, erythrocyte sediment at the bottom of the microplate without a hemagglutination process results in a positive HI test; conversely, hemagglutination in the form of a diffused erythrocyte layer at the bottom of the microplate without an erythrocyte sediment results in a negative outcome. Based on Figure 1, presents the HI test, (A) depicts the absence of hemagglutination, which is defined as erythrocyte sedimentation when the microplate is tilted, and (B) illustrates the occurrence of hemagglutination, which is defined as erythrocyte absence at the microplate well’s bottom. The maximum serum dilution that could prevent hemagglutination against antigens was also used to calculate the HI titer.

There were no discernible changes between groups C-, C+, T1, and T2 when the antibody titer of broiler chickens was examined at 14 days of age, or 7 days after the initial ND vaccination. Analysis by average revealed that all treatment groups’ antibody titers seemed to be moderate and did not yet exceed ND-protective titers (≥7 HI log 2).

On day 35, broiler antibody titers varied (p = 0.010) between groups, indicating that mycotoxin binder may have an impact on the development of ND antibody titers. According to the results of the Duncan test, there were no significant differences between the treatment groups C- with T1, C- with T2, C+ with T1, and T1 with T2, but the C+ with C- and C+ with T2 groups were significantly different, as shown by different letter superscripts in the same column ( Table 1). On the other hand, compared to the average antibody titer of chickens at 14 and 28 days of age, the antibody titer of broilers at 35 days of age increased significantly. The immunological response to the vaccine has only recently started to emerge; the immune system is still in the stage of recognizing the first entered antigen, which accounts for the low mean of antibody titer in 14-day-old broilers (7 days after the initial ND vaccination). The main immune response is the body’s immunological reaction to antigens that initially enter the body. ^22^

**Table 1.** Antibody titers in 14, 28, and 35-day-old broilers.

Treatment group	Replication	Antibody Titers (log 2) (Mean ± SD)		
		14 ^th^ Day	28 ^th^ Day	35 ^th^ Day
C-	5	3.2 ^a^ ± 0.83	4.2 ^a^ ± 0.44	7.4 ^b^ ± 0.89
C+	5	2.8 ^a^ ± 1.09	3.6 ^a^ ± 2.07	5.8 ^a^ ± 0.83
T1	5	2.8 ^a^ ± 0.44	4.4 ^a^ ± 1.14	7.2 ^ab^ ± 1.48
T2	5	3.2 ^a^ ± 1.30	4.0 ^a^ ± 0.70	8.6 ^b^ ± 1.14

^a,b^Different superscripts in the same column indicate significant differences (p < 0.05).

### Histopathological evaluation

Histopathological improvement of the cecum and colon was reported based on reduced mucosal rupture, hemorrhage and necrosis on day 35.

**Figure 2.**
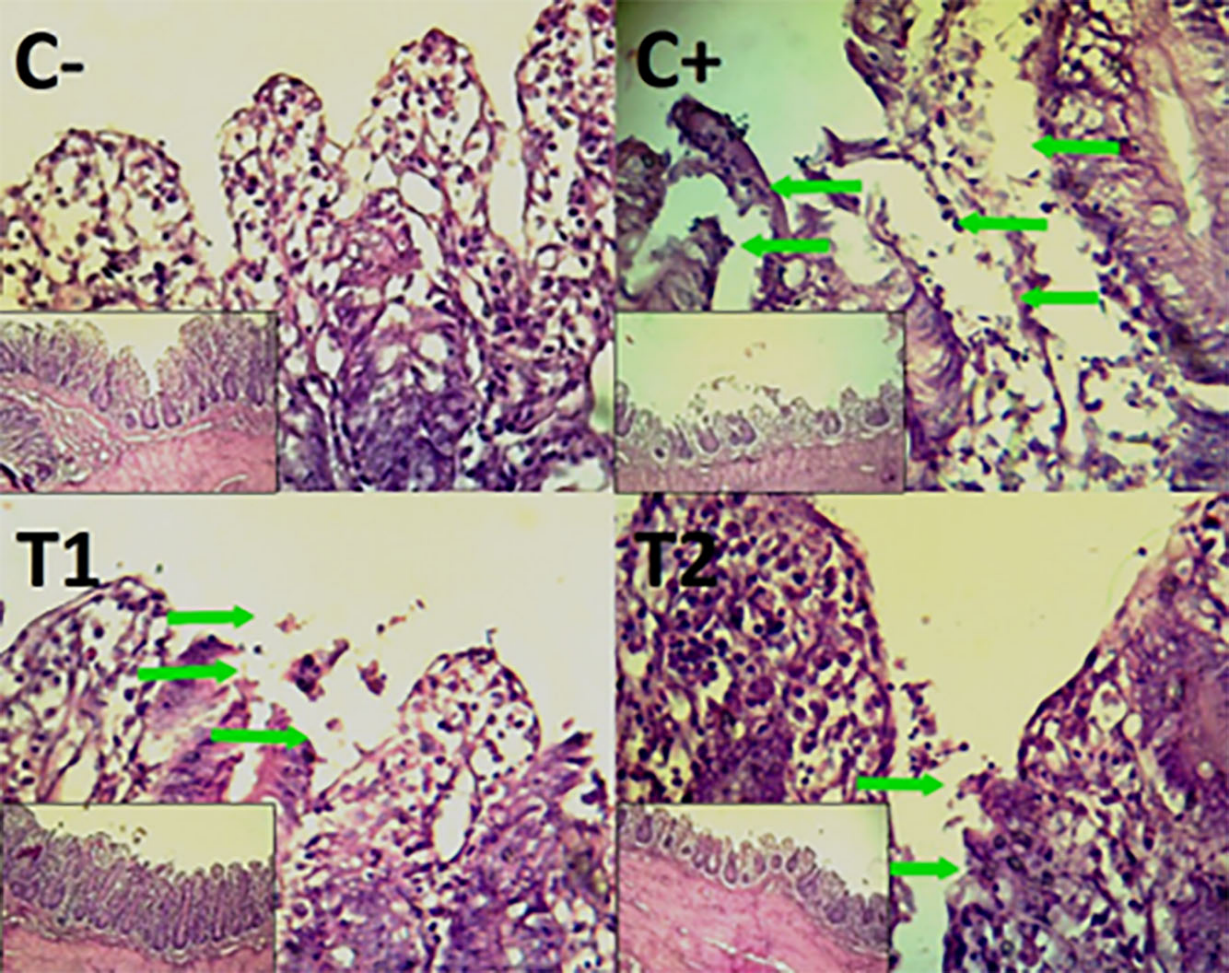
Histopathology of the cecum mucosal rupture ( ➔) using 100× and 400× magnification with HE staining.

**Figure 3.**
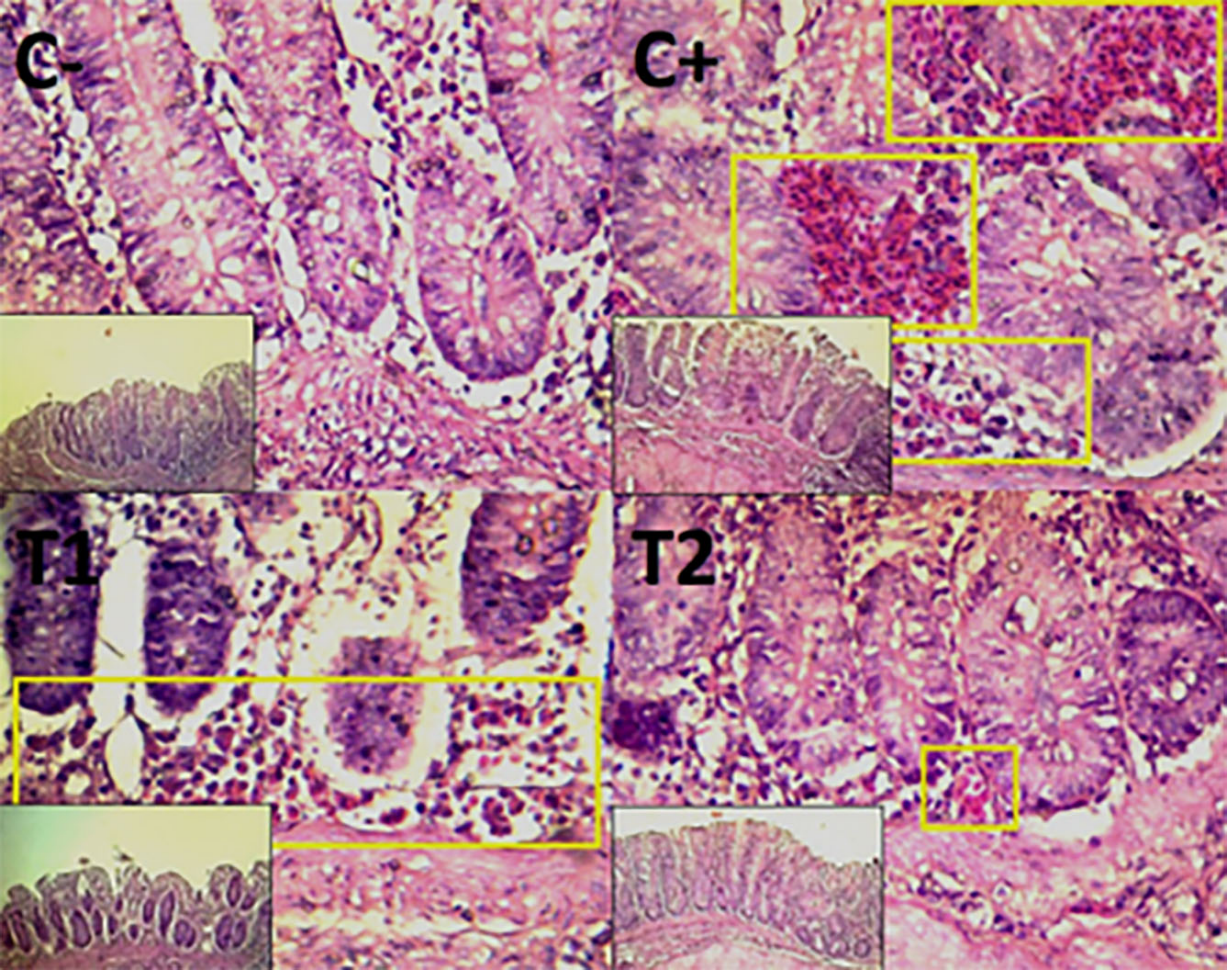
Histopathology of the cecum hemorrhage (yellow area) using 100× and 400× magnification with HE staining.

**Figure 4.**
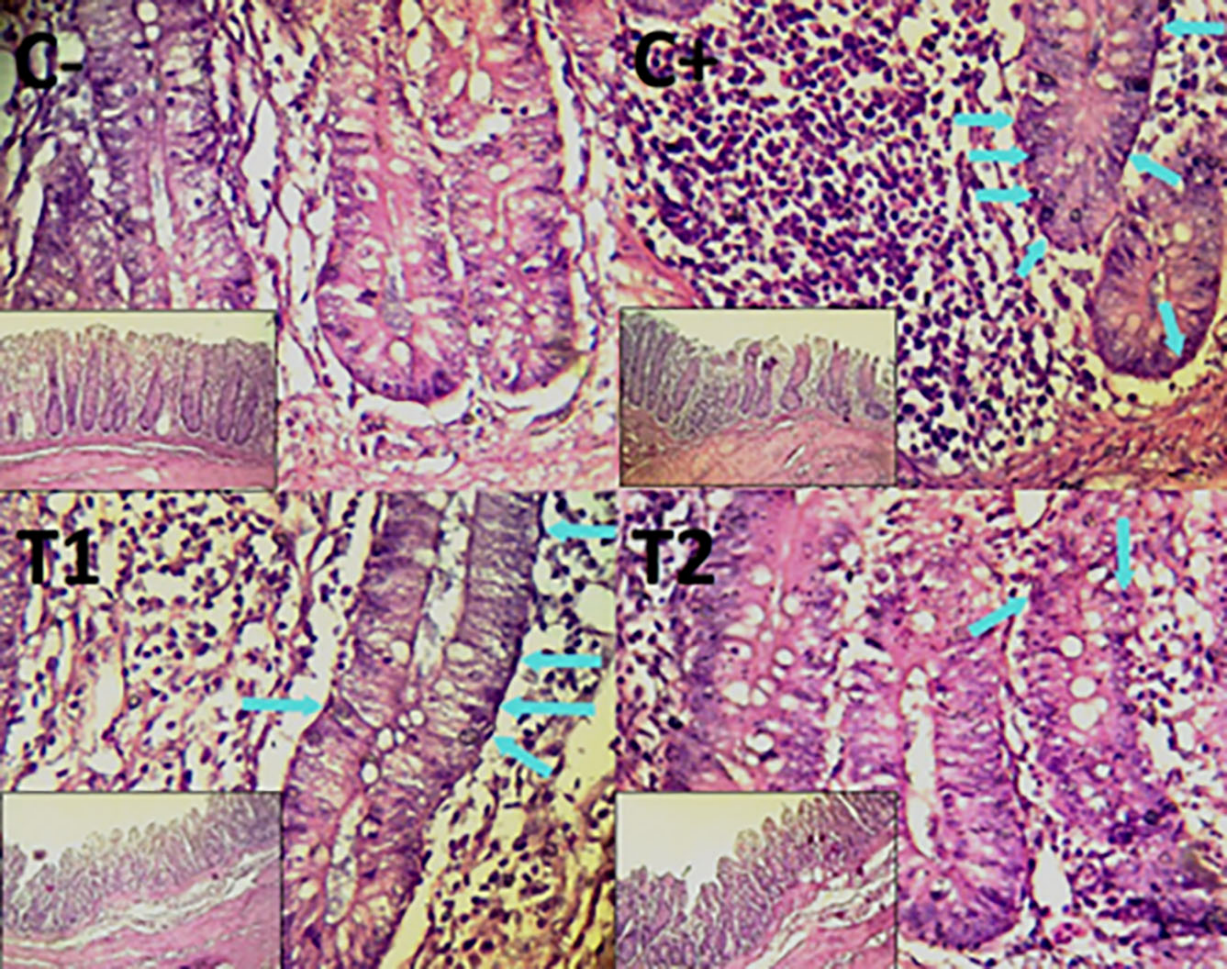
Histopathology of the cecum necrosis ( ➔) using 100× and 400× magnification with HE staining.

The findings of the scoring analysis of the broiler chickens’ mucosal rupture, necrosis, and hemorrhage in the cecum ( Table 2) and colon ( Table 3). The control group (C-), had the lowest mean rank. The C+ treatment group, had the highest mean rank. The T1 treatment group and Treatment group T2 as indicated in Table 2. Furthermore, the T2 group demonstrated a progressive improvement in the histological differences related to mucosal rupture, hemorrhage, and necrosis in the cecum organ ( Figures 2– 4).

**Table 2.** Histopathological evaluation of the cecum at the end of the study.

Treatment group	Parameters (Mean rank)		
	Mucosal rupture	Hemorrhage	Necrosis
C-	3.8 ^c^	4.7 ^c^	5.0 ^c^
C+	17.9 ^a^	17.6 ^a^	17.5 ^a^
T1	13.1 ^b^	13.1 ^b^	12.9 ^b^
T2	7.2 ^c^	6.6 ^c^	6.6 ^c^

^a,b,c^Different superscripts in the same column indicate significant differences (p < 0.05).

**Table 3.** Histopathological evaluation of the colon at the end of the study.

Treatment group	Parameters (Mean rank)		
	Mucosal rupture	Hemorrhage	Necrosis
C-	4.5 ^c^	5.0 ^c^	4.2 ^c^
C+	17.9 ^a^	18.0 ^a^	17.3 ^a^
T1	12.8 ^b^	12.8 ^b^	13.2 ^b^
T2	6.8 ^c^	6.2 ^c^	7.3 ^c^

^a,b,c^Different superscripts in the same column indicate significant differences (p < 0.05).

**Figure 5.**
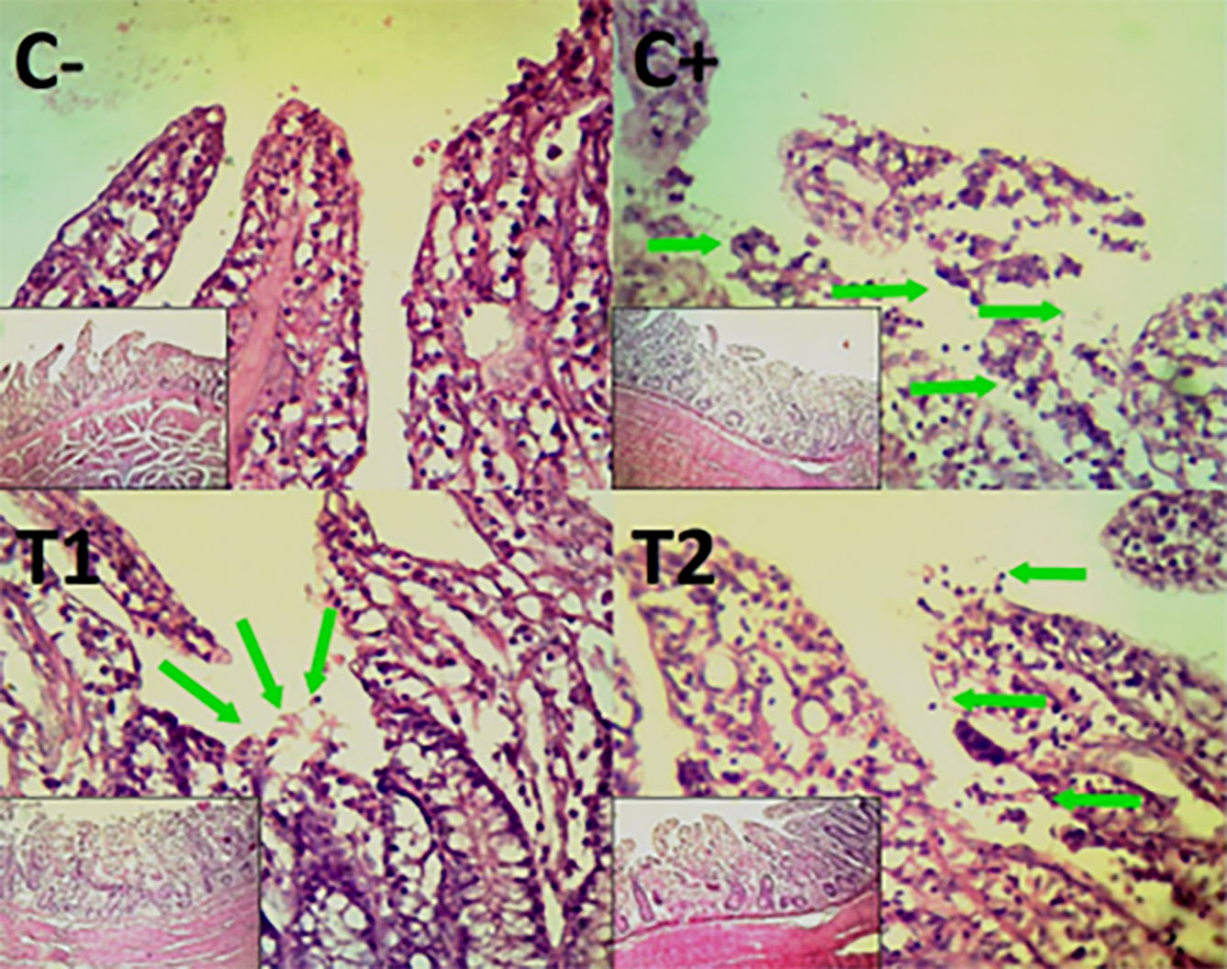
Histopathology of the colon mucosal rupture ( ➔) using 100× and 400× magnification with HE staining.

**Figure 6.**
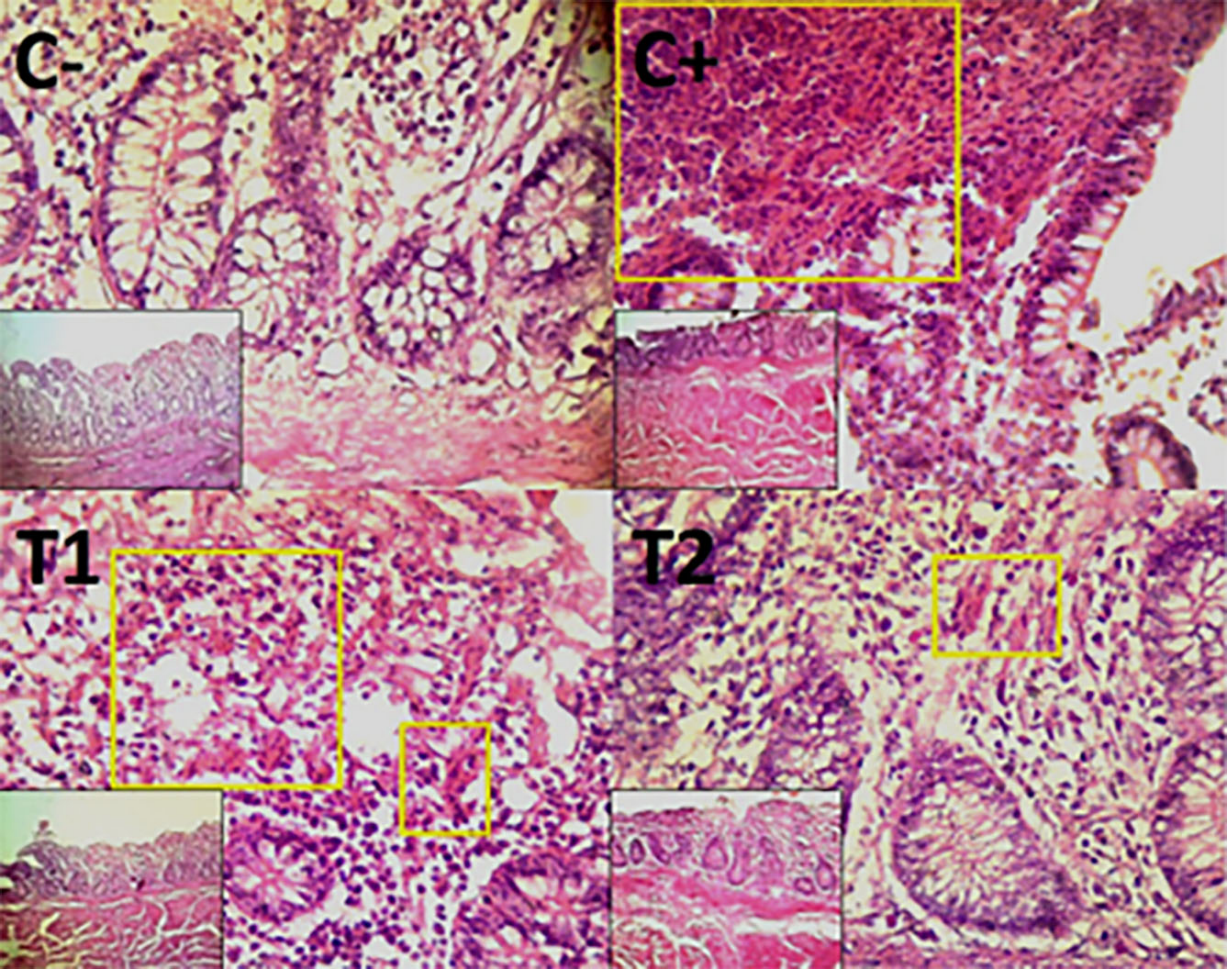
Histopathology of the colon hemorrhage (yellow area) using 100× and 400× magnification with HE staining.

**Figure 7.**
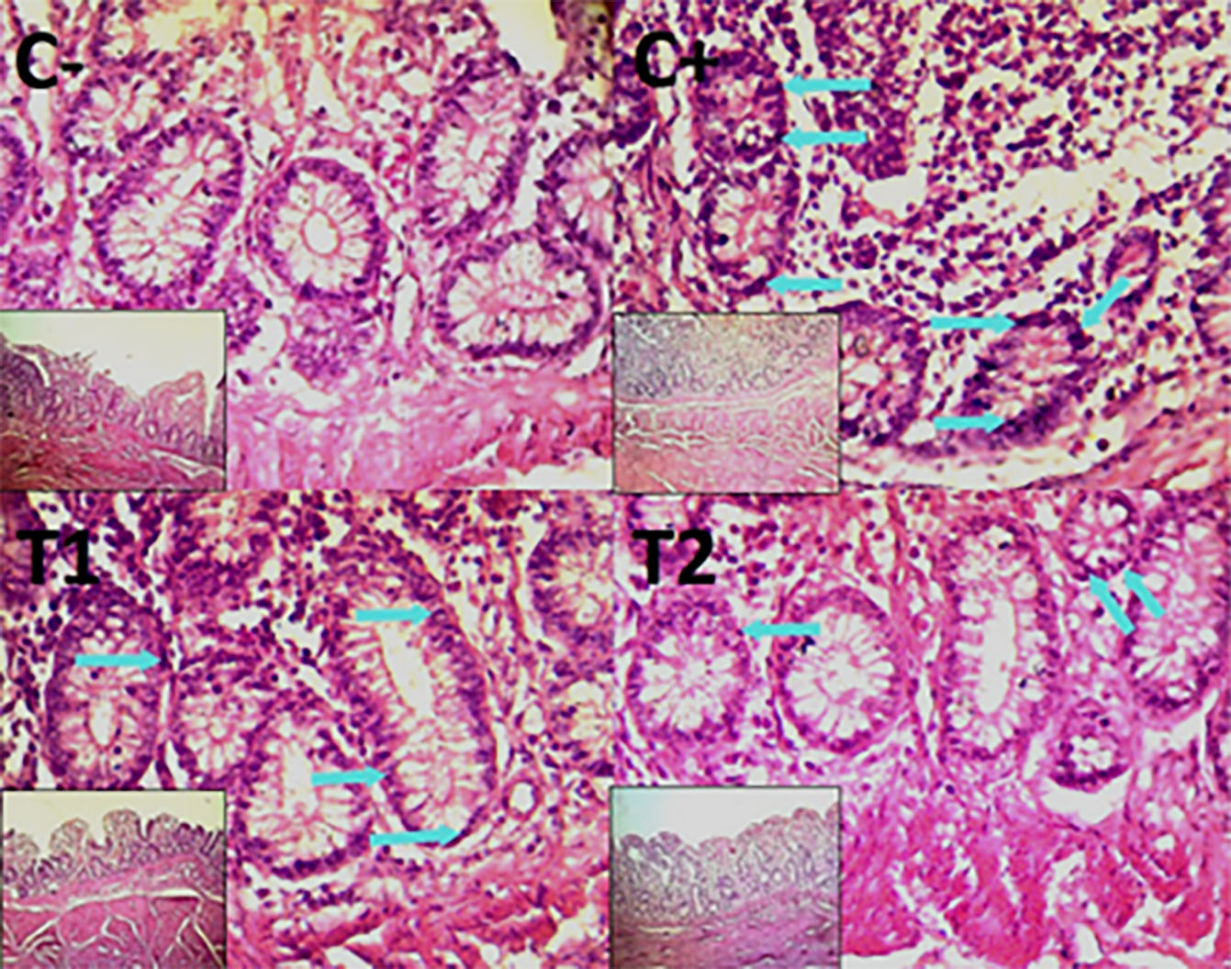
Histopathology of the colon necrosis ( ➔) using 100× and 400× magnification with HE staining.

The control group, treatment group C-, had the lowest mean rank. Treatment group C+, had the highest mean rank. The T1 treatment group. Table 3 shows that the T2 treatment group demonstrated a progressive improvement in the histological differences of mucosal rupture, haemorrhage, and necrosis in the colon organ ( Figures 5– 7).

## Discussion

The primary immune responses that initiated by cell-mediated immunity (CMI) often follow a more gradual course than secondary immunological responses. T cells that serve the role of CMI will develop into effector T cells, or T helper (CD4+) and T cytotoxic (CD8+). Both CD8+ and CD4+ go through three phases in their response to antigens: the expansion phase, the death phase, and the memory phase. ^23^ Seven days after the antigen (organism) enters the body, the organism enters the growth phase, which is followed by the death phase, in which homeostatic processes cause the death of over 90% of active T cells. ^24^

The investigation of antibody titer evaluation in this research performed seven days after booster vaccination in 28-day-old broilers following ND vaccination revealed no significant difference between the C-, C+, T1, and T2 groups. In contrast to the antibody titer at 14 days of age, the average antibody titer in the C-, C+, T1, and T2 groups increased. Immunoglobulin or antibody levels tend to be low seven days after the first vaccination when the HI test is conducted because of this mechanism, which prevents IL-2 produced by CD4+ and CD8+ from signaling B cells to perform maximum cell differentiation. In order to remember and identify antigens to contribute to the secondary immune response during booster immunization, the remaining active T cells will go into the memory phase. ^25^

The development of a secondary immune response could be the cause of the increase in antibody titer at 28 days of age. Booster vaccinations containing the same antigen can accelerate the proliferation and differentiation of B cells by activating memory cells produced from the main immune response. ^26^

In all treatment groups, the average antibody titer appears to have increased, but it is still below protective levels (≥ 7 HI log 2). Because the body’s production of antibodies against the inactive vaccine has not yet reached its maximal titer, this is conceivable. According to a prior study, because inactive vaccines contain an oil adjuvant, vaccination with them will result in the highest antibody titer occurring 14–21 days after the vaccination. ^23^ In order to improve the immune response, adjuvants are substances that are added to vaccines. By slowing down the body’s process of destroying antigens, adjuvants prolong the time that the vaccination comes into touch with macrophages and lymphocytes, which delays the vaccine’s ability to induce the development of antibodies. ^27^

At 35 days of age, the analysis of antibody titers revealed significant differences (p < 0.05) between the C+ group with C- and C+ with T2. In contrast, there was no significant difference between the C- and T1 groups, C- and T2 groups, C+ and T1 groups, and T1 and T2 groups. The broiler group with the lowest average antibody titer among the other treatment groups is C+, which was exposed to AFB1 and OTA without the use of any additional mycotoxin binder agents. According to a prior study, immunotoxic diseases might arise in the body as a result of mycotoxin exposure. ^28^ The primary immune cell apoptotic pathways, autophagy, and oxidative stress will all play a role in the immunotoxic mechanism. These cells include B lymphocytes, dendritic cells, macrophages, neutrophils, and T cells. Moreover, the body experiences immunosuppression, autoimmunity, immunological dysfunction, and hypersensitivity reactions as a result of mycotoxin exposure. ^29^

The impact of chronic mycotoxicosis conditions linked to weight loss, cancer, immunosuppression, and pathological conditions with other slow onset times is known as immunosuppression. In this study, the effect of immunosuppression resulting from mycotoxin exposure on post-vaccination antibody titers has not been observed significantly at 14 and 28 days of age examination. The type, duration, dose, age, and species of the mycotoxin all affect the intensity and symptoms of exposure. If mycotoxins are given to chickens for longer than three to four weeks, the result is chronic mycotoxicosis. ^30, 31^

The analysis of antibody titers at 35 days of age revealed significantly different findings in group C+ with T2 (p < 0.05) in addition to group C+ with C-. T2 is a group of broilers given commercial feed that has been exposed to AFB1 and OTA as well as a mycotoxin binder agent at a dose of 1.6 g/kg feed. In order to prevent the toxicity of mycotoxins from spreading throughout the body, mycotoxin binder agents as feed additives typically function by lowering the bioavailability of mycotoxins while they are still in the gastrointestinal tract. Mycotoxin binders, such as bentonite clay, and mycotoxin modifiers, such as *Trichosporon mycotoxinivorans* yeast, are two key components found in mycotoxin binder agent products. When it comes to removing mycotoxin exposure from the body, the two of them collaborate well. ^32^

As an enterosorbent, bentonite can bind polar mycotoxins like AFB1 in a certain way. It disperses into the gastrointestinal tract’s fluid, becoming negatively charged. This negative charge can then absorb aflatoxins through cation exchange reactions or positive charges from AFB1 dicarbonyl bonds. ^33^ The pH, binder content, mycotoxin kind and dosage, and incubation duration are just a few of the numerous variables that impact an adsorbent’s capacity to interact with mycotoxins. ^34^^,^^35^ Unlike bentonite, the yeast *Trichosporon mycotoxinivorans* has the capacity to decrease the bioavailability of ochratoxin A (OTA) by the hydrolysis of the amide bond structure that links the hydro-isocoumarin ring and the phenylalanine group. Two less hazardous chemicals, L-β-phenylalanine and α-ochratoxin (OTα), will be produced if the amide link is effectively hydrolysed. ^36^^,^^37^

Using mycotoxin binder chemicals as feed additives to bind mix mycotoxin can help prevent uncontrollably contaminated mycotoxin mixes. Using mycotoxin binders, the mix of mycotoxin is detoxified according to its specific features. In this work, the polar toxin known as aflatoxin B1 is detoxified using bentonite, an adsorbent that will boost enzyme activity and prevent exposure to the toxin in the digestive system. Mycotoxin binders function by biotransformation, which is the process of converting mixed mycotoxin into non-toxic metabolites, as opposed to ochratoxin A, which is non-polar. ^37^^,^^38^

Mycotoxin binder is an option that can be utilised as a feed additive in the large intestine, with an ideal dosage of 1.6 g/kg feed. The integrity of the intestinal mucosa will be impacted if the toxicity of mix mycotoxin declines. It will result in fewer goblet cells in the large intestine, which will make the intestinal mucosa less susceptible to injury and mucosal rupture. ^10^

In an attempt to detoxify the toxins created by mixed mycotoxin and lessen their toxicity, a mycotoxin binder is utilized, with an ideal dosage of 1.6 g/kg feed. Aflatoxin B1 will be adsorbed by the mycotoxin binder, but ochratoxin A will be biotransformed. By detoxifying the poison created by mixing mycotoxin, a mycotoxin binder will lower the amount of toxin generated. Lower toxin levels will undoubtedly have an impact on endothelial damage brought on by exposure to mixed mycotoxin; as endothelial damage declines, the endothelium’s role as a mediator of fluid or plasma release that aids in hemostasis will rise. ^10^^,^^12^

As a mycotoxin deactivator for Aflatoxin B1 and Ochratoxin A, mycotoxin binder, with an ideal dosage of 1.6 g/kg feed, is one of the things that can be done. The varied target mycotoxin will determine how the mycotoxin binder functions. Because it has a polar chemical structure and a strong, stable specific bond that will deactivate it, aflatoxin B1 functions as a mycotoxin binder by adsorption. When employing common adsorbents like aluminum silicate, which break down and expand when combined with water, this cannot occur. At pH 3, zeolite will also no longer have its adsorption qualities. The mycotoxin binder in Ochratoxin A, on the other hand, will work as an enzymatic breakdown with biotransformation to break down the mycotoxin into non-toxic metabolites. The combination mycotoxin won’t have a harmful impact, which causes oxidative stress in the form of necrosis or cell death if the toxin exposure has been eliminated. ^12^^,^^21^

## Conclusions

The study’s findings revealed that providing a mycotoxin binder agent to broilers exposed to AFB1 and OTA at a dose of 1.6 g/kg influences the development of antibody titers following ND vaccination and also guards against rupture, hemorrhage, and necrosis in cecum and colon.

## Author contributions

**Erma Safitri, Siti Darodjah Rasad, Goo Jang, and Mitsuhiro Takagi:** Supervision, Research Conceptualization, Methodology and Research Observation, Writing—Original Draft Preparation; **Hery Purnobasuki, Tita Damayanti Lestari, Suzanita Utama, Eka Pramyrtha Hestianah, Mohammad Anam Al Arif, Chairul Anwar Nidom, Sri Mulyati, Sri Hidanah, and Jola Rahmahani:** Curation for Data, Analysis and Formal Investigation, Resources of References, Visualization, Writing—Review and Editing; **Merisa Wahyu Erdhina and Maulida Ilma Sadida:** Curation for Data, Software and Validation; **Martia Rani Tacharina and Muhammad Thohawi Elziyad Purnama:** Curation for Data, Proof Read, Review and Editing.

## Data Availability

Figshare: Database of hemagglutination inhibition test data, histopathology scoring of cecum and colon, and original histopathology figures. https://doi.org/10.6084/m9.figshare.27248592.v2. ^39^ The extended data set contains the following data:
• Antibody titer • Cecum histopathology scoring • Colon histopathology scoring • Histopathology figure of cecum • Histopathology figure of colon Data are available under the terms of the Creative Commons Attribution 4.0 International license (CC-BY 4.0). Figshare: ARRIVE Guidelines 2.0 for Mycotoxin binders study. https://doi.org/10.6084/m9.figshare.27900534.v1. ^40^ Data are available under the terms of the Creative Commons Zero “No rights reserved” data waiver (CC0 1.0 Public domain dedication).

## Software availability

To analyse the quantitative data, SPSS version 26 (IBM Corp., Armonk, NY) was used. If readers are looking for an open-source substitute, JASP ( https://jasp-stats.org/) can be used to conduct comparable analyses.
