# Supplementary material for: Mycotoxin binder for immune and intestinal histopathology ameliorations against Newcastle disease in vaccinated broilers
Source: F1000Res. 2025 Jul 25;13:1518. Originally published 2024 Dec 13. [Version 3] doi: 10.12688/f1000research.158103.3 (PMC12361880; doi:10.12688/f1000research.158103.3)
Supplement: Peer review history

# Reviewer report: Reviewer response for version 3

**DOI:** 10.5256/f1000research.185498.r400455

**Published:** 2025-08-18

**Authors:** Mohammad Miftakhus Sholikin

**Affiliations:** 1 National Research and Innovation Agency (BRIN), Bogor, Indonesia

I had no further comments. Please recheck the names and affiliations. Also, ensure that the bibliography matches the in-text citations. Finally, conduct another round of proofreading to make sure there are no typos or logical errors in the writing.

Is the work clearly and accurately presented and does it cite the current literature?

Yes

If applicable, is the statistical analysis and its interpretation appropriate?

Partly

Are all the source data underlying the results available to ensure full reproducibility?

Yes

Is the study design appropriate and is the work technically sound?

Partly

Are the conclusions drawn adequately supported by the results?

Yes

Are sufficient details of methods and analysis provided to allow replication by others?

Partly

Reviewer Expertise:

Animal Nutrition

I confirm that I have read this submission and believe that I have an appropriate level of expertise to confirm that it is of an acceptable scientific standard.

# Reviewer report: Reviewer response for version 2

**DOI:** 10.5256/f1000research.176969.r394912

**Published:** 2025-07-03

**Authors:** Mohammad Miftakhus Sholikin

**Affiliations:** 1 National Research and Innovation Agency (BRIN), Bogor, Indonesia

The study presents an interesting topic; however, several aspects of the manuscript require revision. The following points highlight necessary improvements:

 Despite these revisions, the research remains compelling. However, one crucial element appears absent from the article, and its inclusion is essential:

 1. The specific type of mycotoxin binder remains unclear.  

 2. The contamination levels of AFB1 and OTA in the treated feed are not explicitly stated.  

 3. The source of the mycotoxins is also not explained.  

 The use of scientific phrases should also be reviewed, as the language currently resembles popular writing rather than scientific standards.

 Suggested revisions:

 1. Abstract: Consider revising the phrase:  

    "An increase in the mean antibody titer compared to days 14 and 28 was observed on day 35."  

    This sentence needs clarification for consistency and precision.

 2. The abbreviation ROS appears to be defined more than once in the main text. Please verify and correct such redundancies.

 3. Suggested rephrasing for scientific accuracy:  

    - Original: "Mycotoxin binder is a big molecular molecule that resembles a chemical sponge and is used to bind mixtures of mycotoxin in an animal’s digestive tract before it is expelled through its faeces."  

    - Suggested: "Mycotoxin binders are typically composed of natural or synthetic adsorbent materials rather than single molecular structures. They function by capturing mycotoxins in the gastrointestinal tract and facilitating their excretion through feces."

 4. The description of Mycofix in the methodology section lacks detail. Clarify its specifications, including key components and possibly the raw material origin.

 5. The study used only 20 samples, with one broiler representing each replicate. Please justify the rationale behind this limited sample size, especially considering the collaboration with several institutions.

 6. In Table 1, the replication statement should not appear in the table content. It is more appropriate as a table note. Moreover, ANOVA values are absent. If treatment effects are not significant, as seen on days 14 and 28 where all superscripts are "a," it is unnecessary to display them.

 7. Tables 2 and 3 do not present ANOVA significance values or measures of variation such as SD, SE, or SEM. Please include these to strengthen data interpretation.

 8. Figure layout and labels overlap, reducing visual clarity. Adjust the formatting to improve readability.

 9. In the Discussion section, avoid repeating statistical significance values such as "(p < 0.05)." The focus should be on interpreting the findings rather than restating results.  

    For example, in the sentence:  

    "At 35 days of age, the analysis of antibody titers revealed significant differences (p < 0.05) between the C+ group with C- and C+ with T2. In contrast, there was no significant difference between the C- and T1 groups, C- and T2 groups, C+ and T1 groups, and T1 and T2 groups."  

    Instead of restating outcomes, explain the potential biological or experimental factors behind the observed patterns.

 10. When mentioning the same species repeatedly, abbreviate it after the first mention. For instance, change *Trichosporon mycotoxinivoran* to *T. mycotoxinivoran* in subsequent mentions.

Is the work clearly and accurately presented and does it cite the current literature?

Yes

If applicable, is the statistical analysis and its interpretation appropriate?

Partly

Are all the source data underlying the results available to ensure full reproducibility?

Yes

Is the study design appropriate and is the work technically sound?

Partly

Are the conclusions drawn adequately supported by the results?

Yes

Are sufficient details of methods and analysis provided to allow replication by others?

Partly

Reviewer Expertise:

Animal Nutrition

I confirm that I have read this submission and believe that I have an appropriate level of expertise to confirm that it is of an acceptable scientific standard, however I have significant reservations, as outlined above.

# Response

**Published:** 2025-07-22

**Authors:** Muhammad Thohawi Elziyad Purnama

**Affiliations:** Airlangga University, Indonesia

Dear

**Dr. Mohammad Miftakhus Sholikin**, National Research and Innovation Agency (BRIN), Bogor, Indonesia

 We would like to express our sincere gratitude to the reviewer for the valuable suggestions and constructive feedback provided on our manuscript. The comments have been instrumental in improving both the content and clarity of our work. We have revised the manuscript accordingly and hope that the changes meet the reviewer’s expectations and enhance the overall scientific contribution of this study.

 ---

 The study presents an interesting topic; however, several aspects of the manuscript require revision. The following points highlight necessary improvements:

 Despite these revisions, the research remains compelling. However, one crucial element appears absent from the article, and its inclusion is essential:

 1. The specific type of mycotoxin binder remains unclear.

**Author Response: **The mycotoxin binder utilized in this study functions not only as an adsorbent but also as a biotransformation agent, converting toxic compounds into non-toxic metabolites. Specifically, the bentonite component effectively binds aflatoxin B1 (AFB1), which possesses a polar molecular structure, whereas ochratoxin A (OTA), being non-polar, requires enzymatic degradation into less toxic forms, a process facilitated by *Trichoderma reesei*. This statement has been reflected in the revised version of the manuscript.

 2. The contamination levels of AFB1 and OTA in the treated feed are not explicitly stated.

**Author Response: **Revision has been implemented in the experimental animal and housing statement.

 3. The source of the mycotoxins is also not explained.

**Author Response: **Revision has been implemented in the experimental animal and housing statement.

 The use of scientific phrases should also be reviewed, as the language currently resembles popular writing rather than scientific standards.

 Suggested revisions:

 1. Abstract: Consider revising the phrase:  

    "An increase in the mean antibody titer compared to days 14 and 28 was observed on day 35."  

    This sentence needs clarification for consistency and precision.

**Author Response: **Revision has been implemented in abstract.

 2. The abbreviation ROS appears to be defined more than once in the main text. Please verify and correct such redundancies.

**Author Response: **Revision has been implemented in the entire manuscript.

 3. Suggested rephrasing for scientific accuracy:  

    - Original: "Mycotoxin binder is a big molecular molecule that resembles a chemical sponge and is used to bind mixtures of mycotoxin in an animal’s digestive tract before it is expelled through its faeces."  

    - Suggested: "Mycotoxin binders are typically composed of natural or synthetic adsorbent materials rather than single molecular structures. They function by capturing mycotoxins in the gastrointestinal tract and facilitating their excretion through feces."

**Author Response: **Revision has been implemented according to recommendation.

 4. The description of Mycofix in the methodology section lacks detail. Clarify its specifications, including key components and possibly the raw material origin.

**Author Response: **Revision has been implemented in experimental animal and housing.

 5. The study used only 20 samples, with one broiler representing each replicate. Please justify the rationale behind this limited sample size, especially considering the collaboration with several institutions.

**Author Response: **The sample size was determined in accordance with ethical clearance guidelines, which mandate the minimal use of experimental animals. In addition to meeting ethical requirements, the sample size was calculated using the Federer formula, *t(n − 1) ≥ 15*, where *t* represents the number of treatment groups and *n* denotes the number of replicates per group. This statement has been reflected in the revised version of the manuscript.

 6. In Table 1, the replication statement should not appear in the table content. It is more appropriate as a table note. Moreover, ANOVA values are absent. If treatment effects are not significant, as seen on days 14 and 28 where all superscripts are "a," it is unnecessary to display them.

**Author Response: **Revision has been implemented according to recommendation.

 7. Tables 2 and 3 do not present ANOVA significance values or measures of variation such as SD, SE, or SEM. Please include these to strengthen data interpretation.

**Author Response: **Revision has been implemented according to recommendation.

 8. Figure layout and labels overlap, reducing visual clarity. Adjust the formatting to improve readability.

**Author Response: **Improvisation of figures and layouts will be assisted by the publisher's technical team.

 9. In the Discussion section, avoid repeating statistical significance values such as "(p < 0.05)." The focus should be on interpreting the findings rather than restating results.  

    For example, in the sentence:  

    "At 35 days of age, the analysis of antibody titers revealed significant differences (p < 0.05) between the C+ group with C- and C+ with T2. In contrast, there was no significant difference between the C- and T1 groups, C- and T2 groups, C+ and T1 groups, and T1 and T2 groups."  

    Instead of restating outcomes, explain the potential biological or experimental factors behind the observed patterns.

**Author Response: **Revision has been implemented according to recommendation in discussion section.

 10. When mentioning the same species repeatedly, abbreviate it after the first mention. For instance, change *Trichosporon mycotoxinivoran* to *T. mycotoxinivoran* in subsequent mentions.

**Author Response: **Revision has been implemented in the entire manuscript.

# Reviewer report: Reviewer response for version 2

**DOI:** 10.5256/f1000research.176969.r393150

**Published:** 2025-07-02

**Authors:** Ammar M. Al-Aalim

**Affiliations:** 1 University of Mosul, Mosul, Iraq

This manuscript deals with the mycotoxin binder effect on the vaccination

 The materials and methods were not explained clearly, and so little information about raising chickens, with little information about how tests are conducted, and also why the author used inaccurate tests like HA and HI, while it could have used the ELISA test to detect antibody titer, which provides more sensitive and accurate results ???

 The results, especially in Figure 1, need to be explained correctly, as well as all statistical analyses must be explained clearly.

Is the work clearly and accurately presented and does it cite the current literature?

Yes

If applicable, is the statistical analysis and its interpretation appropriate?

Partly

Are all the source data underlying the results available to ensure full reproducibility?

Partly

Is the study design appropriate and is the work technically sound?

Yes

Are the conclusions drawn adequately supported by the results?

Yes

Are sufficient details of methods and analysis provided to allow replication by others?

Partly

Reviewer Expertise:

Veterinary Immunology

I confirm that I have read this submission and believe that I have an appropriate level of expertise to confirm that it is of an acceptable scientific standard, however I have significant reservations, as outlined above.

# Response

**Published:** 2025-07-22

**Authors:** Muhammad Thohawi Elziyad Purnama

**Affiliations:** Airlangga University, Indonesia

Dear

**Dr. Ammar M. Al-Aalim**, University of Mosul, Mosul, Iraq

 We would like to express our sincere gratitude to the reviewer for the valuable suggestions and constructive feedback provided on our manuscript. The comments have been instrumental in improving both the content and clarity of our work. We have revised the manuscript accordingly and hope that the changes meet the reviewer’s expectations and enhance the overall scientific contribution of this study.

 ---

 This manuscript deals with the mycotoxin binder effect on the vaccination

 The materials and methods were not explained clearly, and so little information about raising chickens, with little information about how tests are conducted, and also why the author used inaccurate tests like HA and HI, while it could have used the ELISA test to detect antibody titer, which provides more sensitive and accurate results ???

**Author Response: **Revisions have been made to the sections on *Methods*, including *Experimental Animals and Housing*, *Hemagglutination (HA) and Hemagglutination Inhibition (HI) Tests*, and portions of the *Histopathological Evaluation*. The HA and HI tests were employed in this study due to their simplicity, cost-effectiveness, and reliability in measuring antibody titers, expressed in log₂ values.

 The results, especially in Figure 1, need to be explained correctly, as well as all statistical analyses must be explained clearly.

**Author Response: **Additional caption has been provide in the Figure 1 legend. In addition, statistical analysis method statement has been improved properly.

# Reviewer report: Reviewer response for version 2

**DOI:** 10.5256/f1000research.176969.r372034

**Published:** 2025-04-23

**Authors:** Rifat Ullah Khan

**Affiliations:** 1 Khyber Pakhtunkhwa Agricultural University, Khyber Pakhtunkhwa, Pakistan

This study evaluated the protective effects of a mycotoxin binder (1.6 g/kg feed) against aflatoxin B1 and ochratoxin A in broilers vaccinated against Newcastle disease. The binder significantly improved antibody titers and reduced histopathological damage (mucosal rupture, hemorrhage, necrosis) in the cecum and colon, highlighting its potential to mitigate immunosuppressive effects of mycotoxins and enhance poultry health. 

 I have a few concerns and after specific revision, the paper is accepted for publication. Revise the introduction by dividing it into multiple paragraphs for better readability and incorporate the following citations for improved quality:

 Ref: 1,2 

 Additionally, consider whether the sample size is sufficient, as it appears to be quite small. Please also include details on how the virulence of mycotoxins was confirmed. Clarify the conclusion

Is the work clearly and accurately presented and does it cite the current literature?

Yes

If applicable, is the statistical analysis and its interpretation appropriate?

Yes

Are all the source data underlying the results available to ensure full reproducibility?

Yes

Is the study design appropriate and is the work technically sound?

Yes

Are the conclusions drawn adequately supported by the results?

Partly

Are sufficient details of methods and analysis provided to allow replication by others?

Yes

Reviewer Expertise:

Poultry science

I confirm that I have read this submission and believe that I have an appropriate level of expertise to state that I do not consider it to be of an acceptable scientific standard, for reasons outlined above.

## References

[1] : Fabrication of Polydopamine/hemin/TiO2 Composites with Enhanced Visible Light Absorption for Efficient Photocatalytic Degradation of Methylene Blue. *Polymers (Basel)* .2025;17(3) : 10.3390/polym17030311 10.3390/polym17030311 39940513 PMC11820547

[2] : Role of Quercetin in Diabetic Cardiomyopathy. *Plants (Basel)* .2024;14(1) : 10.3390/plants14010025 10.3390/plants14010025 39795285 PMC11722747

# Response

**Published:** 2025-07-22

**Authors:** Muhammad Thohawi Elziyad Purnama

**Affiliations:** Airlangga University, Indonesia

Dear

**Dr. Rifat Ullah Khan**, Khyber Pakhtunkhwa Agricultural University, Khyber Pakhtunkhwa, Pakistan

 We would like to express our sincere gratitude to the reviewer for the valuable suggestions and constructive feedback provided on our manuscript. The comments have been instrumental in improving both the content and clarity of our work. We have revised the manuscript accordingly and hope that the changes meet the reviewer’s expectations and enhance the overall scientific contribution of this study.

 This study evaluated the protective effects of a mycotoxin binder (1.6 g/kg feed) against aflatoxin B1 and ochratoxin A in broilers vaccinated against Newcastle disease. The binder significantly improved antibody titers and reduced histopathological damage (mucosal rupture, hemorrhage, necrosis) in the cecum and colon, highlighting its potential to mitigate immunosuppressive effects of mycotoxins and enhance poultry health. 

 I have a few concerns and after specific revision, the paper is accepted for publication. Revise the introduction by dividing it into multiple paragraphs for better readability and incorporate the following citations for improved quality:

**Author Response: **Revision has been implemented in introduction by dividing it into multiple paragraphs.

 Additionally, consider whether the sample size is sufficient, as it appears to be quite small.

**Author Response: **The determination of sample size was guided by ethical clearance requirements, which emphasize the reduction of experimental animal use to the minimum necessary. In addition to meeting ethical standards, the sample size was calculated using the Federer formula, *t(n − 1) ≥ 15*, where *t* represents the number of treatment groups and *n* the number of replicates per group. This statement has been reflected in the revised version of the manuscript.

 Please also include details on how the virulence of mycotoxins was confirmed. Clarify the conclusion

**Author Response: **The virulence of the mycotoxins was confirmed by the presence of histopathological lesions—specifically mucosal rupture, hemorrhage, and necrosis—in the cecum and colon of the positive control group (C+). This statement has been reflected in the revised version of the manuscript.

**References**

 1. Zhu Z, Zhou S, Tian D, Li GZ, et al.: Fabrication of Polydopamine/hemin/TiO2 Composites with Enhanced Visible Light Absorption for Efficient Photocatalytic Degradation of Methylene Blue. *Polymers (Basel)*. 2025; **17** (3). PubMed Abstract | Publisher Full Text

 2. Mustafa NH, Siti HN, Kamisah Y: Role of Quercetin in Diabetic Cardiomyopathy. *Plants (Basel)*. 2024; **14** (1). PubMed Abstract | Publisher Full Text

**Author Response: **Both references have been added (Ref No. 41 and 42) along with the results of both studies.

# Reviewer report: Reviewer response for version 1

**DOI:** 10.5256/f1000research.173650.r349347

**Published:** 2025-01-06

**Authors:** Agung Irawan

**Affiliations:** 1 Universitas Sebelas Maret, Oregon State University,, Surakarta, Indonesia

**Overall Assessment:**

 The authors evaluated the efficacy of mycotoxin binders on immunity and histopathology of broilers chickens. The topic of the manuscript is interesting and highly relevant to be disseminated. However, I found that in the current form, the manuscript requires substantial revision. Thorough the manuscript, it is poorly written, with significant issues in language, grammar, and coherence. Additionally, the scientific content is not presented in a manner consistent with standard academic practices, which undermines its clarity and impact.

 Here are some major issues for authors’ attention:

- The introduction lacks a logical structure for hypothesis formulation.
- There is a lack of coherence between sentences.
- The narrative is disjointed, making it difficult to follow the authors' rationale.
- The results section contains many unnecessary descriptions that obscure the main findings.
- A more concise and focused presentation of the data is required.
- The discussion is poorly structured and hard to follow.
- The findings are not effectively described, and there is a lack of chronological organization.
- Critical interpretations are not adequately supported by references.
- The authors mention a "hypothesis" in the conclusion but fail to clearly state it in the introduction.
- The manuscript does not adequately discuss or revisit the hypothesis in the discussion section.

For more detailed review, please see below comments:

**#Introduction**

 The introduction is not systematically written, there are too many issues with languages and grammar. Authors need to substantially revise the introduction both grammatically and scientific soundness. Between sentences’ connection is lacking in many parts of paragraphs, some unnecessary information is also found.

 Some information requires more clarity, such as:

- 72% samples tested positive for…. -> please indicate what samples? It is not clear!
- Are the mycotoxin combination that has been shown to have high toxicity -> please be more specific on the toxicity effects and indicate whether the study was in humans or animals (specify the animal)
- There are some issues with the coherences of sentences in the first paragraph, making the reader a bit confused with the main idea of the paragraph. For instance, authors explain some molecular pathways associated with toxicity of AFB1 and OTA, and then jumped to failure of vaccination program etc. A large portion of the paragraph discuss ND vaccination issue while the importance of mycotoxin exposure issues is lost. Although they are relatable, but it is not written systematically. Please review and rewrite this part to be easier to understand.
- At the end of 2 ^nd^ paragraph: plant goods -> what does that mean? I was unfortunately unable to understand. Can the authors please clarify?
- The statement of the first sentence of 3 ^rd^ paragraph is not correct. Fiber digestion in poultry occurs primarily in the gizzard (mechanistic digestion) and only very little portion of the fiber is digested in the large intestine and this does not supply much energy for the bird. Please review this part and rewrite…
- The statement of mycotoxicosis in person is not relevant in this part.
- In the last paragraph, I do not see how the hypothesis was built, there is missing connection between the scientific background, gap of knowledge, and the objective being pursued in this study. Also, *Trichosporon mycotoxinivorans* is mentioned with no reason!

**#Materials and Methods**

- Rpm should be converted into *g *to be more standardized.
- In statistical analysis part, first sentence: followed -> analyzed. Authors mentioned Kruskal Wallis and Mann Whitney tests as well in this part, which one was actually used for the data analysis? Why Mann Whitney was used?

**#Results**

- Please rewrite the results to be more concise. At the present form, there are many unnecessary descriptions. It would be better to consult with other experts or to copyedit the manuscript. Some examples are given below:
- Please remove “as a result” in the first sentence of the section.
- “Based on Figure 1, presents the HI test” -> Figure 1 (A) depicts
- Paragraph 3: The investigation of 35-day-old broiler chickens’ antibody titers revealed statistically different results, with a p-value of 0.010 (p < 0.05). The fact that the value of p < 0.05 suggests that the administration of a mycotoxin binders agent has an impact on the antibody titer after ND vaccination -> Please change to “Antibody titers of broilers on day 35 differed (p = 0.010) between groups, suggesting an effect of mycotoxin binder on ND antibody titers establishment”.

**#Discussion**

 The discussion is hard to follow. Some terms are not specified such as “organism enter the growth phase….” -> it is ambiguous what organism did the authors mean, whether bird or antigen?

 - This paper may be useful as a reference: Putra RP et al. (2024 [Ref-1]) https://link.springer.com/article/10.1007/s11259-023-10199-7 

**#Conclusion: A**uthors mentioned the “hypothesis” but never stated their hypothesis in the introduction and never discussed it.

Is the work clearly and accurately presented and does it cite the current literature?

Partly

If applicable, is the statistical analysis and its interpretation appropriate?

Partly

Are all the source data underlying the results available to ensure full reproducibility?

Yes

Is the study design appropriate and is the work technically sound?

Partly

Are the conclusions drawn adequately supported by the results?

Partly

Are sufficient details of methods and analysis provided to allow replication by others?

Yes

Reviewer Expertise:

Nutritional biochemistry of poultry and ruminants related to feed, feed technology, and feeding.

I confirm that I have read this submission and believe that I have an appropriate level of expertise to confirm that it is of an acceptable scientific standard, however I have significant reservations, as outlined above.

## References

[1] : Protective effects of feed additives on broiler chickens exposed to aflatoxins-contaminated feed: a systematic review and meta-analysis. *Vet Res Commun* .2024;48(1) : 10.1007/s11259-023-10199-7 225-244 10.1007/s11259-023-10199-7 37644237

# Response

**Published:** 2025-01-12

**Authors:** Muhammad Thohawi Elziyad Purnama

**Affiliations:** Airlangga University, Indonesia

Dear reviewers,

 We appreciate your advice on our manuscript. Your suggestions have been addressed and the manuscript has been updated. In our opinion, these changes result in a much better manuscript, and we hope that it is now suitable for publication.

**Overall Assessment:**

 The authors evaluated the efficacy of mycotoxin binders on immunity and histopathology of broilers chickens. The topic of the manuscript is interesting and highly relevant to be disseminated. However, I found that in the current form, the manuscript requires substantial revision. Thorough the manuscript, it is poorly written, with significant issues in language, grammar, and coherence. Additionally, the scientific content is not presented in a manner consistent with standard academic practices, which undermines its clarity and impact.

 Here are some major issues for authors’ attention:

- The introduction lacks a logical structure for hypothesis formulation
**Author Response:** Revision has been implemented, We changed the hypothesis formulation with aims the research
- There is a lack of coherence between sentences
**Author Response:** Revision has been implemented
- The narrative is disjointed, making it difficult to follow the authors' rationale.
**Author Response:** Revision has been implemented
- The results section contains many unnecessary descriptions that obscure the main findings.
**Author Response:** Revision has been implemented
- A more concise and focused presentation of the data is required.
**Author Response:** Revision has been implemented
- The discussion is poorly structured and hard to follow.
**Author Response:** Revision has been implemented
- The findings are not effectively described, and there is a lack of chronological organization. **Author Response:** Revision has been implemented
- Critical interpretations are not adequately supported by references.
**Author Response:** Revision has been implemented
- The authors mention a "hypothesis" in the conclusion but fail to clearly state it in the introduction.
**Author Response:** We have revised the word “hypothesis” in conclusion
- The manuscript does not adequately discuss or revisit the hypothesis in the discussion section.
**Author Response:** Revision has been implemented

For more detailed review, please see below comments:

**#Introduction**

 The introduction is not systematically written, there are too many issues with languages and grammar. Authors need to substantially revise the introduction both grammatically and scientific soundness. Between sentences’ connection is lacking in many parts of paragraphs, some unnecessary information is also found

**Author Response:** Revision has been implemented

 Some information requires more clarity, such as:

- 72% samples tested positive for…. -> please indicate what samples? It is not clear!
**Author Response:** Revision has been implemented
- Are the mycotoxin combination that has been shown to have high toxicity -> please be more specific on the toxicity effects and indicate whether the study was in humans or animals (specify the animal)
**Author Response:** We have rewrited
- There are some issues with the coherences of sentences in the first paragraph, making the reader a bit confused with the main idea of the paragraph. For instance, authors explain some molecular pathways associated with toxicity of AFB1 and OTA, and then jumped to failure of vaccination program etc. A large portion of the paragraph discuss ND vaccination issue while the importance of mycotoxin exposure issues is lost. Although they are relatable, but it is not written systematically. Please review and rewrite this part to be easier to understand.
**Author Response:** We have revised in paragraph 1 & 2 introduction
- At the end of 2 ^nd^ paragraph: plant goods -> what does that mean? I was unfortunately unable to understand. Can the authors please clarify?
**Author Response:** We have changed the statement with this : The digestive system has the highest risk of exposure since animal feed is the first point in the food chain where mixed mycotoxins can enter directly through grains crop
- The statement of the first sentence of 3 ^rd^ paragraph is not correct. Fiber digestion in poultry occurs primarily in the gizzard (mechanistic digestion) and only very little portion of the fiber is digested in the large intestine and this does not supply much energy for the bird. Please review this part and rewrite…
**Author Response:** The process of digesting and absorbing dietary fibre in chickens is significantly aided by the gizzard  (mechanistic digestion) and ends in the large intestine. (This sentence is at the end of the first paragraph)
- The statement of mycotoxicosis in person is not relevant in this part.
**Author Response:** We have changed the statement with this : Mycotoxicosis is poisoning that occurs when animals eat feed containing mycotoxins, where the toxins from these mycotoxins cannot be tolerated by the body
- In the last paragraph, I do not see how the hypothesis was built, there is missing connection between the scientific background, gap of knowledge, and the objective being pursued in this study. Also, *Trichosporon mycotoxinivorans* is mentioned with no reason! **Author Response:** We have changed the hypothesis with aim statement. Also about *Trichosporon mycotoxinivorans*, we have rewrited

**#Materials and Methods**

- Rpm should be converted into *g *to be more standardized.
**Author Response:** Revision has been implemented
- In statistical analysis part, first sentence: followed -> analyzed. Authors mentioned Kruskal Wallis and Mann Whitney tests as well in this part, which one was actually used for the data analysis? Why Mann Whitney was used?
**Author Response:** Revision has been implemented

**#Results**

- Please rewrite the results to be more concise. At the present form, there are many unnecessary descriptions. It would be better to consult with other experts or to copyedit the manuscript. Some examples are given below:
- Please remove “as a result” in the first sentence of the section.
**Author Response:** Revision has been implemented
- “Based on Figure 1, presents the HI test” -> Figure 1 (A) depicts
**Author Response:** We have revised

Paragraph 3: The investigation of 35-day-old broiler chickens’ antibody titers revealed statistically different results, with a p-value of 0.010 (p < 0.05). The fact that the value of p < 0.05 suggests that the administration of a mycotoxin binders agent has an impact on the antibody titer after ND vaccination -> Please change to “Antibody titers of broilers on day 35 differed (p = 0.010) between groups, suggesting an effect of mycotoxin binder on ND antibody titers establishment”.

**Author Response:** I've changed it according to reviewer's suggestion

**#Discussion**

 The discussion is hard to follow. Some terms are not specified such as “organism enter the growth phase….” -> it is ambiguous what organism did the authors mean, whether bird or antigen?

**Author Response:** We have rewrited

 - This paper may be useful as a reference: Putra RP et al. (2024 [Ref-1]) https://link.springer.com/article/10.1007/s11259-023-10199-7 

 Putra RP, Astuti D, Respati AN, Ningsih N, et al.: Protective effects of feed additives on broiler chickens exposed to aflatoxins-contaminated feed: a systematic review and meta-analysis. *Vet Res Commun*. 2024; **48** (1): 225-244

**Author Response:** We have added in Reference number 35

**#Conclusion: **Authors mentioned the “hypothesis” but never stated their hypothesis in the introduction and never discussed it.

**Author Response:** We have deleted the word “hypothesis” in conclusion

# Reviewer report: Reviewer response for version 1

**DOI:** 10.5256/f1000research.173650.r349350

**Published:** 2024-12-26

**Authors:** Essam S Soliman

**Affiliations:** 1 Food Control and Hygiene, King Salman International University, Ras Sedr, South Sinia, Egypt; 2 Suez Canal University, Ismailia, Egypt

Manuscript **F1KR00CDE F1R-VER173650-R** entitled “ **Implementation of mycotoxin binders to enhance immune response and large intestine histopathology against Newcastle disease in vaccinated broiler chickens fed naturally contaminated diet with mixed mycotoxins**”. **Please notice the following:**

**General view:** The manuscript highlighted that a toxin binder dose of 1.6 g/kg can increase antibody titer and histopathology of cecum and colon in broiler chickens after ND vaccination fed with mycotoxin-contaminated feed. The manuscript was expressed using moderate English and grammar. A certain degree of copyediting & proofreading, proper display of the introduction, and declaring some explanations of the materials and methods have to be made to achieve better readability & understanding, and publication value.

**Title:** Very long. Preferred to be modified for clarity and simplification into “ **Mycotoxin Immune and Intestinal Histopathology Ameliorations** **Against Newcastle Disease** **in Vaccinated Broilers**”.

**Abstract:** Please notice the following:

 1. Clear to a great extent.

 2. Some modifications must be made to enhance the readability and understanding of the text.

**Keywords:** Re-arrange in alphabetical order as follows: “Aflatoxin, Antibody titer, Broilers, Food production, Ochratoxin, Toxin binders”.

**Introduction: **Please notice the following:

1. Improperly arranged into four paragraphs.
2. The introduction has to be re-arranged into three paragraphs only i.e. 1. Introduction 2. Significance of the study, and 3. Aim of the study.
3. Some modifications must be made to enhance the readability and understanding of the text.

**The aim**: Clear to a certain extent. Some modifications must be made to enhance the readability and understanding of the text.

**Materials and methods:** Please notice the following:

 1. MUST provide details on the microclimatic conditions used for rearing the experimental broilers such as ambient temperature, relative humidity, feeding system (manual/automated), watering system (mechanism and source & type of water), lighting program (Color, duration, and intensity), ventilation system, rodentproof, flyproof, cleaning procedures (Provide the active principle of the detergent used), disinfection protocol (Provide the active principle of the disinfectant used), and treatment applied if any.

 2. Provide the total number of blood samples collected by the end of the study and specify their types (With or without anticoagulant).

 3. Provide a reference for the Euthanasia technique used in broilers.

 4. Explain how the carcasses were disposed of hygienically after sampling.

 5. Specify the number of the histopathological samples collected.

 6. Provide the statistical model exhausted in the statistical analysis.

 7. Some modifications must be made to enhance the readability and understanding of the text.

**Results:** Please notice the following:

 1. MUST not list the P-value for the non-significant results.

 2. Do not list the details of the treatment groups all over and use the symbols instead.

 3. Reduce the illustration of numbers as much as possible as they are listed in tables and express them in your own words.

 4. Some modifications must be made to enhance the readability and understanding of the text.

**Discussion:** Please notice the following:

 1. Clear to a certain extent.

 2. A moderate degree of speculation and comparison was used.

 3. Some modifications must be made to enhance the readability and understanding of the text.

**Conclusion:** Clear and concise.

**Authors’ contributions: **Clear.

**Acknowledgment: **Clear.

**References:** Sufficient as 62.1% (23 out of 37) were published in the last five years.

**Tables:** Properly designed and well organized.

**Figures:** Properly designed and well organized.

Is the work clearly and accurately presented and does it cite the current literature?

Yes

If applicable, is the statistical analysis and its interpretation appropriate?

Yes

Are all the source data underlying the results available to ensure full reproducibility?

Yes

Is the study design appropriate and is the work technically sound?

Yes

Are the conclusions drawn adequately supported by the results?

Yes

Are sufficient details of methods and analysis provided to allow replication by others?

No

Reviewer Expertise:

1. Preventive medicine and prophylactics of animals (Large and small), poultry, and fish. 2. Biosafety and biosecurity measures, plans, and strategies in animal and poultry farms. 3. Green environment and waste management and reuse. 4. Combating means of infectious and contagious diseases. 5. Air and water quality, and environmental sanitation. 6. Epidemiology of infectious and contagious disease and measurement of disease occurrence.

I confirm that I have read this submission and believe that I have an appropriate level of expertise to confirm that it is of an acceptable scientific standard, however I have significant reservations, as outlined above.

# Response

**Published:** 2025-01-12

**Authors:** Muhammad Thohawi Elziyad Purnama

**Affiliations:** Airlangga University, Indonesia

Dear reviewers,

 We appreciate your advice on our manuscript. Your suggestions have been addressed and the manuscript has been updated. In our opinion, these changes result in a much better manuscript, and we hope that it is now suitable for publication.

**General view:** The manuscript highlighted that a toxin binder dose of 1.6 g/kg can increase antibody titer and histopathology of cecum and colon in broiler chickens after ND vaccination fed with mycotoxin-contaminated feed. The manuscript was expressed using moderate English and grammar. A certain degree of copyediting & proofreading, proper display of the introduction, and declaring some explanations of the materials and methods have to be made to achieve better readability & understanding, and publication value.

 Title: Very long. Preferred to be modified for clarity and simplification into “Mycotoxin Immune and Intestinal Histopathology Ameliorations Against Newcastle Disease in Vaccinated Broilers”.

**Author Response:** Yes, We agree to changed the title modification according to the reviewer's suggestion

**Abstract:** Please notice the following:

 1. Clear to a great extent. Author Response: Revision has been implemented.

 2. Some modifications must be made to enhance the readability and understanding of the text.

**Author Response:** We have revised in text

**Keywords:** Re-arrange in alphabetical order as follows: “Aflatoxin, Antibody titer, Broilers, Food production, Ochratoxin, Toxin binders”.

**Author Response:** Yes, We agree to re-arranged in alphabetical, according to the reviewer's suggestion

**Introduction: **Please notice the following:

1. Improperly arranged into four paragraphs.
2. The introduction has to be re-arranged into three paragraphs only i.e. 1. Introduction 2. Significance of the study, and 3. Aim of the study.
3. Some modifications must be made to enhance the readability and understanding of the text. The aim: Clear to a certain extent. Some modifications must be made to enhance the readability and understanding of the text.
**Author Response:** We have revised in text

**Materials and methods:** Please notice the following:

 1. MUST provide details on the microclimatic conditions used for rearing the experimental broilers such as ambient temperature, relative humidity, feeding system (manual/automated), watering system (mechanism and source & type of water), lighting program (Color, duration, and intensity), ventilation system, rodentproof, flyproof, cleaning procedures (Provide the active principle of the detergent used), disinfection protocol (Provide the active principle of the disinfectant used), and treatment applied if any. **Author Response:** We have revised in text

 --> The five steps of preparation before acclimatization of day-old chicks (DOC) are as follows: 1. Clean and remove all physical materials from a chicken cage; 2. Wash and clean all equipment and the cage with soap and water, using scraping; 3. Spray liquid disinfectant on all equipment and the cage and close the cage for a week; 4. Spray solid disinfectant (lime) and mix it with water in the cage and close the cage for a week; 5. Fumigate with permanganate and formalin, close the cage, as this can irritate; and finally, wait a week before DOC arrives.

 --> A total of 20 DOC broiler chickens Cobb strain were reared for 35 days as final stock following broiler rearing standards. Rearing standards for broiler experiments include: basic ventilation system (open house), rodent and flyproof, cleaning and disinfection protocol (five steps before DOC arrived), ambient temperature (18 to 24°C), relative humidity (50 to 70%), manual feeding system, watering system (manual, ad libitum, and groundwater), lighting program (light color at 20 lux intensity for the first 7 days and 17-20 hours duration), and lighting program.

 2. Provide the total number of blood samples collected by the end of the study and specify their types (With or without anticoagulant).

**Author Response:** We have revised in text

 --> A total of 1 mL blood samples without anti-coagulant were collected through the brachial vein in 20 broiler chickens aged 14, 28, and 35 days. Blood was then allowed until collected serum and transferred into an Ependorf tube.

 3. Provide a reference for the Euthanasia technique used in broilers.

**Author Response:** Reference number 18

 4. Explain how the carcasses were disposed of hygienically after sampling.

**Author Response:** The process of incineration involves burning carcasses or residues at temperatures as high as 850 °C to create an inorganic ash. All infectious agents are anticipated to be destroyed by the treatment. Ash usually makes about 1–5% of the initial volume of the carcass, though this will vary depending on the type of incinerator, the method, the fuel, and the species of animal.

 5. Specify the number of the histopathological samples collected.

**Author Response:** After the cecum and colon from 20 broilers were collected

 6. Provide the statistical model exhausted in the statistical analysis.

**Author Response:** Revision has been implemented

 7. Some modifications must be made to enhance the readability and understanding of the text.

**Author Response:** We have revised in text

**Results:** Please notice the following:

 1. MUST not list the P-value for the non-significant results.

**Author Response:** We have deleted in text

 2. Do not list the details of the treatment groups all over and use the symbols instead.

**Author Response:** We have revised

 3. Reduce the illustration of numbers as much as possible as they are listed in tables and express them in your own words.

**Author Response:** Revision has been implemented.

 4. Some modifications must be made to enhance the readability and understanding of the text.

**Author Response:** Revision has been implemented.

**Discussion:** Please notice the following:

 1. Clear to a certain extent.

**Author Response:** Revision has been implemented.

 2. A moderate degree of speculation and comparison was used.

**Author Response:** Revision has been implemented.

 3. Some modifications must be made to enhance the readability and understanding of the text.

**Author Response:** Revision has been implemented.

**Conclusion:** Clear and concise.

**Author Response:** Thank you for your recommendation.

**Authors’ contributions: **Clear.

**Author Response:** Thank you for your recommendation.

**Acknowledgment: **Clear.

**Author Response:** Thank you for your recommendation.

**References:** Sufficient as 62.1% (23 out of 37) were published in the last five years.

**Author Response:** Now 65,7 % (25 out of 38)

**Tables:** Properly designed and well organized.

**Author Response:** Thank you for your recommendation.

**Figures:** Properly designed and well organized.

**Author Response:** Thank you for your recommendation.
